# Effect of intrathecal fentanyl on dexmedetomidine requirement for sedation during spinal anesthesia: a randomized controlled trial

**DOI:** 10.12701/jyms.2026.43.47

**Published:** 2026-07-09

**Authors:** Ki Beom Park, Ji-Yong Yeom, Juhee Min, Hyeon Seung Yi, Gaeul Hur, Eun Kyung Choi

**Affiliations:** 1 Department of Anesthesiology and Pain Medicine, Keimyung University Dongsan Hospital, Keimyung University School of Medicine, Daegu, Korea; 2 Department of Anesthesiology and Pain Medicine, Yeungnam University College of Medicine, Daegu, Korea

**Keywords:** Anesthesia, Deep sedation, Dexmedetomidine, Fentanyl, Spinal anesthesia

## Abstract

**Background:**

Adequate sedation during spinal anesthesia requires careful titration to avoid hemodynamic instability and respiratory complications. We hypothesized that intrathecal fentanyl, used as an adjunct to neuraxial anesthesia, would reduce dexmedetomidine requirements for conscious sedation. This study evaluated the effects of intrathecal fentanyl on the total dose of dexmedetomidine during spinal anesthesia.

**Methods:**

Sixty-four patients undergoing knee or ankle arthroscopy under spinal anesthesia were randomly assigned to two groups. The fentanyl group received 0.5% bupivacaine 10 mg with fentanyl 20 μg, whereas the control group received bupivacaine 10 mg with saline. After confirmation of sensory block, dexmedetomidine was infused at 1 μg/kg over 10 minutes and maintained at 0.1–1 μg/kg/hour to maintain a bispectral index (BIS) of 70 to 80. The primary outcome was the total dose of dexmedetomidine. Secondary outcomes included BIS values, Modified Observer’s Assessment of Alertness/Sedation (MOAA/S) scores, mean blood pressure, and heart rate at predefined time points.

**Results:**

The total dose of dexmedetomidine did not differ significantly between the groups. The MOAA/S scores were significantly affected by group, time, and group-by-time interactions (*p*=0.003, *p*<0.001, and *p*=0.007, respectively). The BIS values were influenced by group (*p*=0.026) and time (*p*<0.001), but not by group-by-time interactions (*p*=0.413). Exploratory analyses suggested deeper sedation in the fentanyl group during the early phase of dexmedetomidine infusion. Hemodynamic variables were comparable between the groups.

**Conclusion:**

Intrathecal fentanyl did not significantly reduce dexmedetomidine requirements during spinal anesthesia, although it was associated with deeper sedation at several time points.

## Introduction

Conscious sedation during spinal anesthesia is commonly used to reduce patient anxiety and enhance overall comfort. It also facilitates surgical procedures by improving patient cooperation. Achieving adequate sedation requires careful selection of a sedative with a wide safety margin and precise dosing to avoid complications, such as hemodynamic instability and respiratory depression.

Dexmedetomidine, a selective α_2_-adrenoreceptor agonist, is increasingly used as a sedative in neuraxial anesthesia. It provides effective sedation and analgesia, improved hemodynamic stability, and minimal respiratory depression [1-3]. Additionally, dexmedetomidine has demonstrated protective effects against postoperative delirium [4] and has been shown to prolong both the duration and quality of spinal anesthesia [5,6]. However, its sympatholytic properties and potential for deep sedation may lead to adverse effects including hypotension, bradycardia, and respiratory depression [7].

When administered intrathecally with a local anesthetic, fentanyl enhances the quality and duration of spinal anesthesia and improves perioperative analgesia. Previous studies suggested that intrathecal fentanyl may reduce the requirement for propofol during conscious sedation under spinal anesthesia [8,9]. Lee et al. [9] reported that intrathecal fentanyl lowered the effect-site concentration of propofol used for conscious sedation during spinal anesthesia, and Kim et al. [8] demonstrated its dose-sparing effect on propofol sedation, as measured by the cerebral state index. However, whether this potential sedative-sparing effect can be extended to dexmedetomidine remains uncertain because dexmedetomidine has distinct pharmacologic characteristics, including α_2_-adrenergic agonism, slower titratability, and intrinsic analgesic-sedative properties.

Based on these considerations, we hypothesized that intrathecal fentanyl reduces dexmedetomidine requirements or alters sedation depth during spinal anesthesia. Therefore, this study aimed to evaluate the effects of intrathecal fentanyl on the total dose of dexmedetomidine and sedation depth in patients undergoing surgery under spinal anesthesia.

## Methods

**Ethics statement:** This study received approval from the Institutional Review Board (IRB) of Yeungnam University Hospital (IRB No: 2017-06-036, approval date: October 17, 2017) and was registered with the Clinical Research Information Service (CRIS; https://cris.nih.go.kr; KCT0005162, registration date: June 4, 2020). Written informed consent was obtained from all participants before enrollment.

### 1. Patients and treatment

We enrolled 64 patients classified as American Society of Anesthesiologists physical status of I to II. Patients were excluded if they had contraindications to spinal anesthesia, cardiovascular disease, known allergies or sensitivities to anesthetic agents, or morbid obesity (body mass index, ≥30 kg/m^2^). Eligible participants underwent lower-extremity orthopedic procedures, including knee and ankle arthroscopy.

Using a computer-generated randomization program, we assigned patients to one of two groups: the BO group, 0.5% bupivacaine 10 mg with 0.4 mL saline and the BF group, 0.5% bupivacaine 10 mg with 20 μg fentanyl. Upon arrival in the operating room, we applied standard monitoring and attached a bispectral index (BIS) sensor before spinal anesthesia. Two anesthesiologists conducted the study; one prepared and administered the spinal anesthetic, while the other monitored the sedation depth and patient status intraoperatively. Both the assessors and patients remained blinded to the group allocation until study completion.

For the spinal procedure, the patients were placed in the lateral decubitus position. Lumbar puncture was performed at the L3 to L4 or L4 to L5 interspace using a 25-gauge spinal needle. We assessed the sensory block level using a pinprick test and confirmed its stability through three consecutive evaluations. To maintain BIS values between 70 and 80 during surgery, we titrated the dexmedetomidine infusion accordingly [10]. Dexmedetomidine hydrochloride (Precedex 100 μg/mL; Hospira Inc., Lake Forest, IL, USA) was diluted to 4 μg/mL in a 50 mL solution relative to the patient’s weight. Following confirmation of the sensory block level 20 minutes after spinal anesthesia, the dexmedetomidine infusion was initiated with a loading dose of 1 μg/kg administered over 10 minutes, followed by a maintenance infusion of 0.1 to 1.0 μg/kg/hour. The infusion rate was titrated to maintain a target BIS of 70 to 80. When BIS values deviated from the target range, the infusion rate was adjusted within the predefined dosing range according to continuous BIS monitoring, observed sedation depth, and the attending anesthesiologist’s clinical judgment. Dose adjustments were performed according to a predefined study protocol, based on continuous BIS monitoring. We recorded BIS values and assessed sedation using the Modified Observer's Assessment of Alertness/Sedation (MOAA/S) scale at five time points: immediately before dexmedetomidine infusion (T0) and 30, 45, 60, and 90 minutes after the start of infusion (T30, T45, T60, and T90, respectively).

Intravenous ephedrine 4 mg was administered when the mean blood pressure (MBP) dropped below 60 mmHg or the systolic arterial pressure fell below 90 mmHg. Nicardipine 0.5 mg was administered intravenously as a bolus when the MBP exceeded 100 mmHg or the systolic arterial pressure rose above 170 mmHg. If the heart rate (HR) fell below 45 beats/minute, atropine 0.5 mg was administered. All the patients received oxygen via a facial mask at a rate of 5 L/minute. In cases of hypoxia (oxygen saturation, <90%), we provided ventilatory support.

The primary outcome was the total dose of dexmedetomidine administered from the initiation of infusion until the end of sedation. The total dose of dexmedetomidine was calculated in μg. Secondary outcomes included BIS values, MOAA/S, MBP, and HR recorded at five time points (T0, T30, T45, T60, and T90); the incidence of bradycardia, hypotension, and hypertension requiring pharmacological treatment during sedation; and the time to discharge after arrival in the post-anesthesia recovery unit (recovery time).

We calculated the sample size based on pilot study data showing that patients in the BO group required 120±32 μg (mean±standard deviation [SD]) of dexmedetomidine. Assuming a 20% reduction in the total dose of dexmedetomidine in the BF group, with an SD of 32 μg, a statistical power of 80%, and a two-sided α of 0.05, at least 28 patients per group were required. Considering an anticipated dropout rate of 15%, 32 patients were enrolled in each group.

### 2. Statistical analysis

The analysis was conducted using IBM SPSS ver. 25 (IBM Corp., Armonk, NY, USA). Continuous variables, including the total dose of dexmedetomidine, were compared between the groups using the Student *t*-test. The incidences of hypotension, hypertension, bradycardia, and desaturation were compared using the chi-square test. Data are presented as mean±SD, median (interquartile range), or number (%).

Repeated measures continuous variables (BIS, MBP, and HR) were analyzed using linear mixed-effects models, with group, time, and group-by-time interactions as fixed effects, and subject as a random effect. MOAA/S scores were analyzed using a generalized linear mixed-effects model with the same fixed- and random-effects structure. These models were used to account for the within-subject correlations across repeated measurements. A two-sided *p*-value of <0.05 was considered statistically significant.

## Results

A total of 64 patients were enrolled in the study, and none were excluded or lost to follow-up (Fig. 1). The demographic characteristics did not differ significantly between the two groups (Table 1). Sedation duration was comparable, with no statistically significant difference (*p*=0.125) (Table 1). Sensory block levels were also similar between the groups, with a median level of T10 (8–10) for both groups (*p*=0.619) (Table 1).

The total dexmedetomidine dose administered during sedation was lower in the BF group (122.0±44.4 μg) than in the BO group (137.2±50.8 μg); however, this difference was not statistically significant (*p*=0.216) (Table 2).

The incidences of bradycardia (requiring atropine), hypotension (requiring ephedrine), and hypertension (requiring nicardipine) did not differ significantly between the groups. The MOAA/S scores were significantly influenced by group (*p*=0.003), time (*p*<0.001), and group-by-time interaction (*p*=0.007) in the generalized linear mixed-effects model analysis (Table 3).

The BIS values were significantly influenced by group (*p*=0.026) and time (*p*<0.001), whereas the group-by-time interaction was not significant (*p*=0.413), indicating similar temporal patterns between the two groups. Exploratory pairwise comparisons demonstrated lower BIS values in the BF group than in the BO group at T30 and T45 (Fig. 2). Similarly, MBP and HR changed significantly over time (both *p*<0.001). However, no significant group effects or group-by-time interaction effects were observed for either variable, indicating similar hemodynamic trajectories between the two groups (Fig. 3).

## Discussion

This study demonstrated that the addition of intrathecal fentanyl to bupivacaine did not significantly reduce the dose of dexmedetomidine required for conscious sedation during spinal anesthesia. However, exploratory analyses of the secondary outcomes suggested deeper sedation in the fentanyl group during the early phase of dexmedetomidine infusion. Hemodynamic parameters showed no significant differences between the groups at any time point.

Adequate sedation during spinal anesthesia is essential for patient comfort and optimal surgical conditions. Conscious sedation, which is defined as a depressed level of consciousness with preserved responsiveness to verbal commands, is typically preferred because it minimizes the risk of hemodynamic and respiratory complications. To achieve this state during neuraxial anesthesia, various sedatives such as propofol and midazolam have been widely used in clinical practice. However, excessive administration of these agents may increase morbidity due to adverse effects, including hypotension, bradycardia, and respiratory depression. To mitigate these risks, intrathecal additives to local anesthetics have been used to supplement sedation and reduce the required doses of systemic agents [8,9].

Neuraxial additives to local anesthetics are known to enhance the duration and quality of spinal anesthesia [11,12]. Among these, fentanyl is the most used intrathecal opioid and numerous studies support its facilitatory effects on spinal block quality [13,14]. Notably, some studies have indicated that intrathecal fentanyl exerts a dose-sparing effect on hypnotic agents used for sedation. For example, Kim et al. [8] found that adding 25 μg of intrathecal fentanyl to 12.5 mg of bupivacaine reduced the propofol dose required for adequate sedation during spinal anesthesia. Similarly, Lee et al. [9] reported that 25 μg of intrathecal fentanyl combined with 10 mg of bupivacaine lowered the optimal effect-site concentration of propofol. Unlike propofol, dexmedetomidine induces sedation via α_2_-adrenergic mechanisms and exhibits slower pharmacodynamic titration characteristics. Therefore, the sedative interactions observed with propofol in previous studies may not be directly generalizable to dexmedetomidine sedation protocols.

However, to the best of our knowledge, no studies have evaluated the sedative-sparing effects of intrathecal fentanyl in the context of dexmedetomidine infusion during spinal anesthesia. Therefore, we hypothesized that intrathecal fentanyl reduces the dose of dexmedetomidine required for conscious sedation. Based on dose-ranging studies of intrathecal administration [15,16], we selected a regimen of 20 μg fentanyl combined with 10 mg of hyperbaric bupivacaine, reflecting the current standard practice. Despite this rationale, our findings did not demonstrate a dexmedetomidine-sparing effect under the study protocol. Two factors could explain this outcome. First, the relatively strong sedative profile and delayed pharmacodynamic characteristics of dexmedetomidine may have limited our ability to detect subtle differences attributable to intrathecal fentanyl under the present study conditions. In support of this interpretation, Park et al. [17] reported that intrathecal fentanyl did not significantly enhance the duration or quality of spinal anesthesia when dexmedetomidine was concurrently administered for sedation. Second, the selected dose of intrathecal fentanyl may have been insufficient to elicit a measurable reduction in dexmedetomidine requirement.

One notable finding of this study was that despite the titration of dexmedetomidine to maintain BIS values between 70 and 80, exploratory analyses suggested lower BIS values and deeper sedation in the BF group during the early phase of dexmedetomidine infusion. Several factors could explain this discrepancy. First, dexmedetomidine exhibits delayed pharmacodynamic characteristics compared to other intravenous sedatives, which may result in transient oversedation despite adjustment of infusion rates [18]. Second, BIS monitoring has an intrinsic processing delay, limiting its ability to reflect real-time changes in sedation depth. Previous studies have demonstrated that BIS values may lag actual clinical sedation status during dexmedetomidine administration [19,20]. Third, intrathecal fentanyl may exert a synergistic sedative interaction with dexmedetomidine through the combined modulation of central nervous system activity, thereby enhancing the sedation depth without necessarily reducing the total dexmedetomidine requirement.

Importantly, dexmedetomidine administration remained consistent with the predefined titration protocol, and no protocol deviations occurred. Therefore, the transient oversedation suggested by lower BIS values and deeper sedation scores during the early phase of dexmedetomidine infusion is more likely attributable to the delayed pharmacodynamic characteristics of dexmedetomidine, inherent processing delay of BIS monitoring, and potential pharmacodynamic interactions between intrathecal fentanyl and dexmedetomidine rather than inadequate protocol adherence. Given the absence of a statistically significant difference in the primary outcome, these observations should be regarded as exploratory secondary findings rather than as definitive evidence of a clinically meaningful dexmedetomidine-sparing effect of intrathecal fentanyl.

Sedation depth was assessed using MOAA/S and BIS monitoring. MOAA/S scores evaluate clinical responsiveness, whereas BIS provides continuous, objective monitoring. Previous studies demonstrated a correlation between these parameters during dexmedetomidine sedation [21].

This study has several limitations that should be considered when interpreting the findings. First, only one intrathecal fentanyl regimen (20 μg combined with 10 mg hyperbaric bupivacaine) was evaluated. It is possible that this dose was insufficient to produce a detectable reduction in the dexmedetomidine requirement during sedation. Additional studies investigating different fentanyl dosages or alternative neuraxial regimens may help clarify any potential sedative-sparing effects. Second, maintaining a predefined BIS target during dexmedetomidine sedation may be challenging because of the drug’s relatively slow pharmacodynamic profile and the inherent processing delay associated with BIS monitoring [19]. Consequently, temporary deviations below the intended BIS range may have occurred despite continuous infusion adjustments throughout the procedure. Third, detailed respiratory variables (e.g., respiratory rate and end-tidal carbon dioxide), fentanyl-related adverse effects (e.g., pruritus, postoperative nausea, and vomiting), and patient satisfaction were not prospectively collected or assessed. Although no episodes of desaturation occurred during the study period, the potential influence of these factors on respiratory safety, sedation quality, and patient experience could not be fully evaluated. Fourth, although blinding was attempted for both patients and outcome assessors, the clinical effects of intrathecal fentanyl could have been recognized during intraoperative management, introducing the possibility of performance or assessment bias. Fifth, the variability in the total dose of dexmedetomidine observed in the final dataset was greater than that expected from the pilot study used for sample size estimation. This increased variability may have reduced the power to detect modest between-group differences. Therefore, the absence of a statistically significant difference in the primary outcome should not be interpreted as evidence of equivalence between the two groups. Larger studies are required to detect smaller treatment effects and to estimate between-group differences more precisely. Finally, this investigation was conducted at a single institution and included patients who were relatively healthy and underwent lower-extremity surgery under spinal anesthesia. Therefore, the generalizability of these findings to a broader surgical population and different clinical settings may be limited.

In conclusion, intrathecal fentanyl administration did not significantly reduce dexmedetomidine requirements during spinal anesthesia. Although lower BIS values and deeper sedation were observed during the early phase of dexmedetomidine infusion, these secondary findings should be interpreted with caution because the primary outcome was not significantly different between the groups. Further, adequately powered studies are required to determine the clinical significance of these findings.

## Figures and Tables

**Fig. 1.**
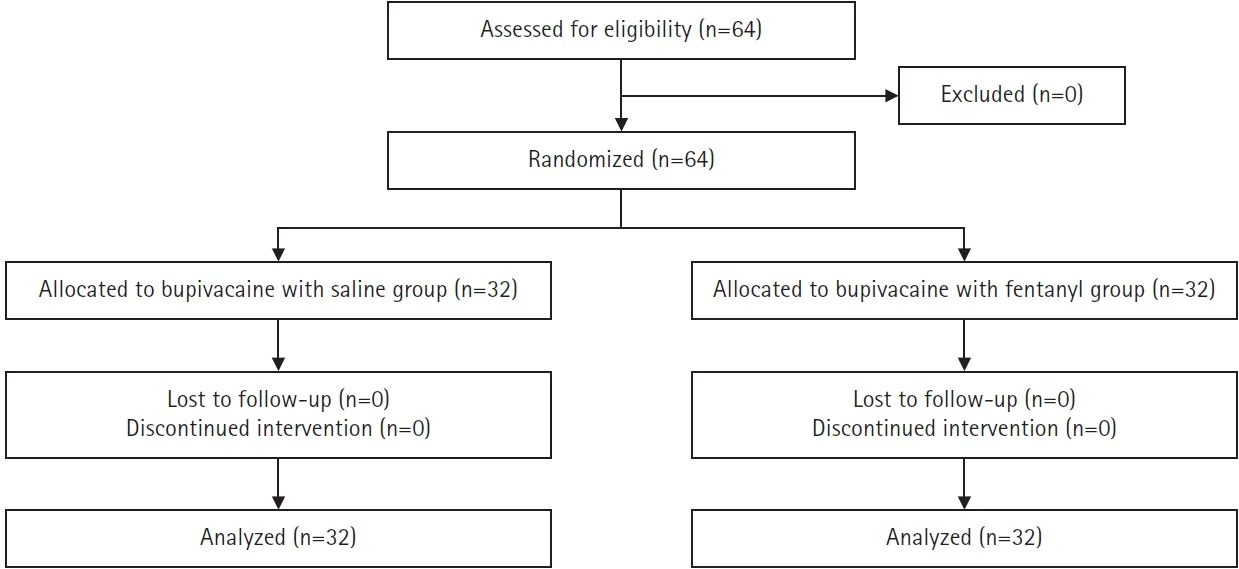
Flow diagram.

**Fig. 2.**
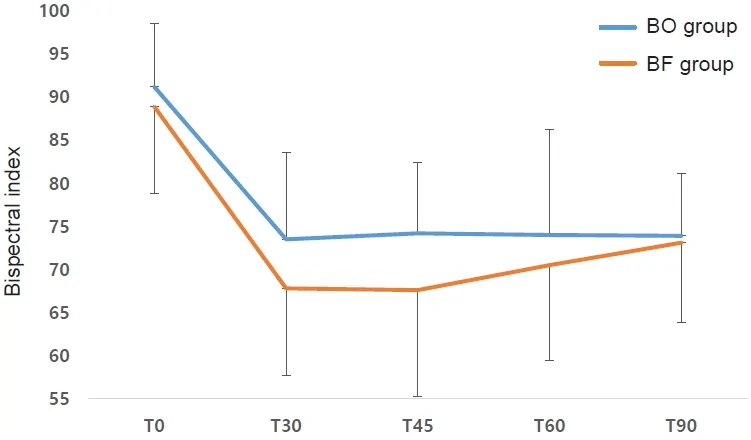
Changes in bispectral index values over time in the BO group (0.5% bupivacaine with saline) and the BF group (0.5% bupivacaine with fentanyl). T0 indicates immediately before dexmedetomidine infusion, and T30, T45, T60, and T90 indicate 30, 45, 60, and 90 minutes after the start of dexmedetomidine infusion, respectively. Data are presented as mean±standard deviation. Statistical analysis was performed using a linear mixed-effects model.

**Fig. 3.**
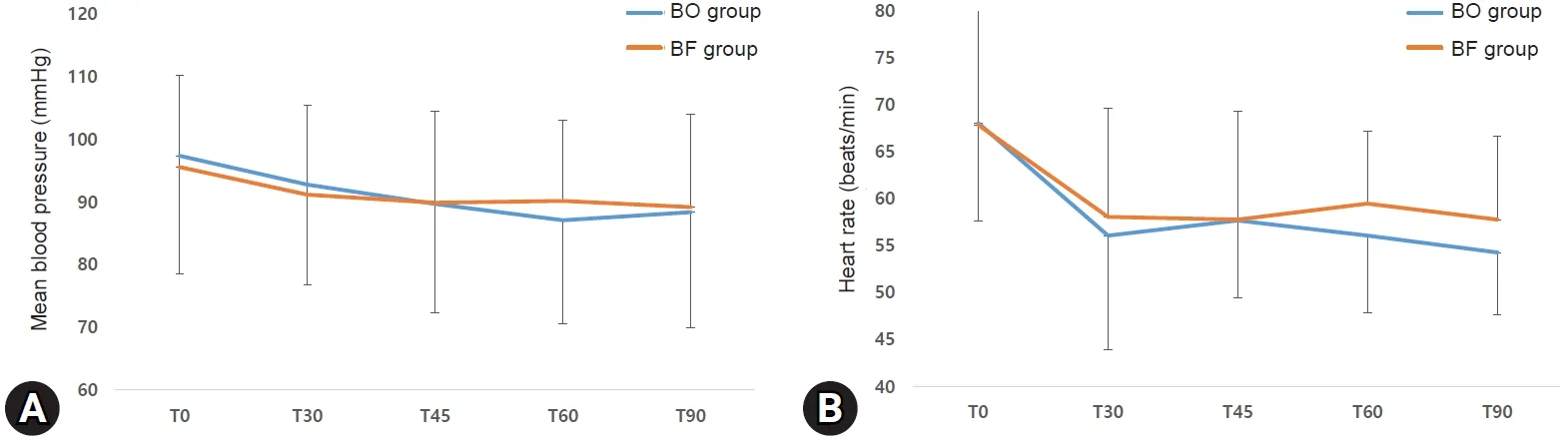
Changes in (A) mean blood pressure and (B) heart rate over time in the BO group (0.5% bupivacaine with saline) and the BF group (0.5% bupivacaine with fentanyl). T0 indicates immediately before dexmedetomidine infusion, and T30, T45, T60, and T90 indicate 30, 45, 60, and 90 minutes after the start of dexmedetomidine infusion, respectively. Data are presented as mean±standard deviation. Statistical analysis was performed using a linear mixed-effects model.

**Table 1.** Demographic data

Characteristic	BO group	BF group	*p*-value
No. of patients	32	32	
Age (yr)	48.5±12.8	50.4±11.6	0.529
Height (cm)	160.0±11.7	164.1±10.3	0.236
Weight (kg)	68.9±19.5	68.7±14.2	0.708
Sex, male:female	13:19	14:18	0.800
Sensory block level	10 (8–10)	10 (8–10)	0.619
Sedation time (min)	94.3±27.7	87.0±42.1	0.125
Operation time (min)	79.0±27.0	78.4±43.8	0.417
Recovery time (min)	28.9±22.7	34.5±13.5	0.231

Values are presented as number, mean±standard deviation, or median (interquartile range). BO group, 0.5% bupivacaine with saline; BF group, 0.5% bupivacaine with fentanyl.

**Table 2.** Dexmedetomidine consumption and sedation-related adverse events

Variable	BO group (n=32)	BF group (n=32)	*p*-value
Total dexmedetomidine consumption (μg)	137.2±50.8	122.0±44.4	0.216
Bradycardia	6 (18.7)	6 (18.7)	>0.999
Hypotension	0 (0)	0 (0)	>0.999
Hypertension	1 (3.1)	2 (6.2)	0.350
Desaturation	0 (0)	0 (0)	>0.999

Values are presented as mean±standard deviation or number (%). BO group, 0.5% bupivacaine with saline; BF group, 0.5% bupivacaine with fentanyl.

**Table 3.** Modified Observer's Assessment of Alertness/Sedation Scale during sedation

Time	BO group (n=32)	BF group (n=32)	*p*-value	
			Time (group)	Interaction (G×T)
T0	5.00 (2.00–5.00)	5.00 (4.00–5.00)	<0.001	0.007
T30	3.00 (2.00–4.00)	2.00 (1.00–3.00)	(0.003)	
T45	3.00 (2.25–4.00)	2.00 (1.00–3.00)		
T60	3.00 (2.00–4.75)	2.00 (2.00–4.00)		
T90	3.00 (2.00–4.00)	2.00 (2.00–4.00)		

Values are presented as median (interquartile range). BO group, 0.5% bupivacaine with saline; BF group, 0.5% bupivacaine with fentanyl; T0, just before dexmedetomidine infusion; T30, 30 minutes after dexmedetomidine infusion start; T45, 45 minutes after dexmedetomidine infusion start; T60, 60 minutes after dexmedetomidine infusion start; T90, 90 minutes after dexmedetomidine infusion start. *p*-values for group, time, and group-by-time (G×T) interaction were obtained using a generalized linear mixed-effects model.
